# Maternal blood contamination of collected cord blood can be identified using DNA methylation at three CpGs

**DOI:** 10.1186/s13148-017-0370-2

**Published:** 2017-07-25

**Authors:** Alexander M. Morin, Evan Gatev, Lisa M. McEwen, Julia L. MacIsaac, David T. S. Lin, Nastassja Koen, Darina Czamara, Katri Räikkönen, Heather J. Zar, Karestan Koenen, Dan J. Stein, Michael S. Kobor, Meaghan J. Jones

**Affiliations:** 1 Centre for Molecular Medicine and Therapeutics, BC Children’s Hospital, Department of Medical Genetics, University of British Columbia, 950 W 28th Ave, Vancouver, BC V5Z 4H4 Canada; 2 Department of Psychiatry and Mental Health, South African Medical Research Council (SAMRC) Unit on Anxiety and Stress Disorders, University of Cape Town, Groote Schuur Hospital, J2, Anzio Road, Observatory, Cape Town, South Africa; 3 Max Planck Institute of Psychiatry, Department of Translational Research in Psychiatry, Kraepelinstraße 2-10, 80804 Munich, Germany; 4 Department of Psychology and Logopedics, Faculty of Medicine, University of Helsinki, P.O.Box 63, 00014 Helsinki, Finland; 5 Department of Paediatrics, MRC Unit on Child and Adolescent Health, University of Cape Town, Room 513 ICH Building Red Cross Children’s Hospital Klipfontein Road, Cape Town, South Africa; 6 Department of Epidemiology, Harvard T. H. Chan School of Public Health, 677 Huntington Avenue, Kresge Building, 505, Boston, MA 02115 USA; 7 Human Early Learning Partnership, University of British Columbia, 2208 East Mall, Vancouver, BC 02115 Canada

**Keywords:** Cord blood, Contamination, DNA methylation, 450K, Genotyping, Maternal blood, Blood banking

## Abstract

**Background:**

Cord blood is a commonly used tissue in environmental, genetic, and epigenetic population studies due to its ready availability and potential to inform on a sensitive period of human development. However, the introduction of maternal blood during labor or cross-contamination during sample collection may complicate downstream analyses. After discovering maternal contamination of cord blood in a cohort study of 150 neonates using Illumina 450K DNA methylation (DNAm) data, we used a combination of linear regression and random forest machine learning to create a DNAm-based screening method. We identified a panel of DNAm sites that could discriminate between contaminated and non-contaminated samples, then designed pyrosequencing assays to pre-screen DNA prior to being assayed on an array.

**Results:**

Maternal contamination of cord blood was initially identified by unusual X chromosome DNA methylation patterns in 17 males. We utilized our DNAm panel to detect contaminated male samples and a proportional amount of female samples in the same cohort. We validated our DNAm screening method on an additional 189 sample cohort using both pyrosequencing and DNAm arrays, as well as 9 publically available cord blood 450K data sets. The rate of contamination varied from 0 to 10% within these studies, likely related to collection specific methods.

**Conclusions:**

Maternal blood can contaminate cord blood during sample collection at appreciable levels across multiple studies. We have identified a panel of markers that can be used to identify this contamination, either post hoc after DNAm arrays have been completed, or in advance using a targeted technique like pyrosequencing.

**Electronic supplementary material:**

The online version of this article (doi:10.1186/s13148-017-0370-2) contains supplementary material, which is available to authorized users.

## Background

Neonatal blood from the umbilical cord at the time of delivery is increasingly being collected for both research and medical purposes. In research, interest in the developmental origins of health and disease has made cord blood a popular choice for genetic, epigenetic, and environmental studies [1]. Cord blood has several physiological differences from adult blood, such as the presence of nucleated red blood cells and fetal hemoglobin, and is an excellent window into the in utero environment, free of confounding post-natal exposures [2, 3]. Medically, cord blood is banked for transplantation as a source of progenitor cells for replenishing the hematopoietic system [4]. Cord blood can be collected after caesarian or vaginal delivery, either preceding or following delivery of the placenta. Both processes typically involve venipuncture of the umbilical artery and collection into a blood bag by gravity [4]. Problems can arise when the collected cord blood becomes contaminated with other cells, most frequently maternal white blood cells [5, 6]. In some cases, maternal blood cells may enter fetal circulation through the placenta. Previous studies have shown that such contamination can occur relatively frequently, estimated at 2–20% of collected samples, but it makes up a very small fraction of fetal blood, with ~10^−4^ to 10^−5^ fetal nucleated cells estimated as maternal [7–10]. This small amount of contamination should have negligible effects on the assessment of DNA or RNA. However, contamination in larger amounts, which could occur through mixing of blood during collection, is of greater concern.

Previous techniques for identifying larger amounts of maternal contributions to collected cord blood have included PCR on highly variable mini satellites or specific polymorphic alleles and fluorescent in situ hybridization (FISH) or TaqMan assay to detect two X chromosomes [7, 9, 11]. Neither technique is universally unambiguous, as mother/child pairs may not be informative for targeted genetic variants, and FISH or TaqMan analysis can only be performed on male children, as they differentiate XX maternal cells from XY child cells [5, 7–9, 11, 12].

DNA methylation (DNAm) is another potential method by which to identify maternal contamination of cord blood, as it is highly different between newborns and adults [13, 14]. DNAm is an epigenetic mark where a methyl group is covalently bound to DNA, primarily at CpG dinucleotides. It is stable under a variety of collection and storage methods, and often employed to identify epigenetic patterns associated with specific environmental or developmental exposures [15–17]. If present at considerable amounts, maternal contamination of cord blood is of concern to studies of DNAm data, as it could mask signals from cord blood or introduce signals present in the maternal blood. This contamination would be differentially observable in male and female children. Since the X chromosome has highly distinct male- and female-specific patterns of DNAm, XX blood from mothers would be more apparent when mixed with XY male children than XX females.

In this study, we initially observed a high proportion of cord blood samples evidently contaminated with maternal blood in the quality control phase of an epigenome-wide association study. Using DNAm data from the genome-wide Illumina 450K array, we created a method by which to identify contaminated samples using 10 CpGs that correctly discriminated contamination status. We also showed that a subset of three CpGs were sufficient for screening DNA using pyrosequencing. While it cannot accurately predict the proportion of contamination, this process is capable of detecting levels that appreciably affect the output of common methods for assessment of DNA methylation. This method can be used to pre-screen prior to running the samples on a DNAm array, or in cases where it is important to identify maternal contamination, such as cord blood banking.

## Results

### Detection of maternal contamination

**Fig. 1:**
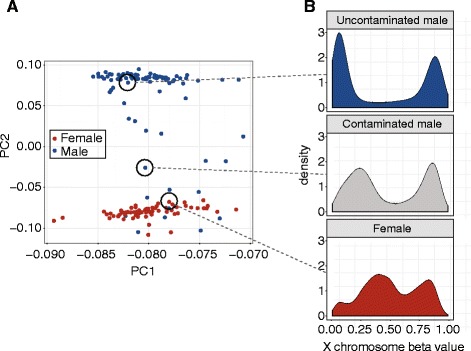
Principal component and X chromosome DNA methylation (DNAm) patterns revealed maternal blood contamination in cord blood. **a** Plotting the first two principal components of 450K DNAm data identified a number of male samples with DNAm patterns similar to female participants or intermediate between male and female. **b** Examining the distribution of X chromosome DNAm beta values in these samples revealed that the intermediate male samples clearly showed patterns indicative of a mixture of male (*top*) and female (*bottom*) distributions

Our first indication of potential maternal contamination of cord blood came from unusual patterns in the DNAm data during quality control. Quality control MDS plots of un-normalized data showed 17 of 86 male participants’ DNAm profiles clustered with female children or in between male and female, which was confirmed by plotting principal components 1 and 2 (Fig. 1a). Investigating the X and Y chromosome probes prior to probe filtering and normalization in more detail, we observed that these male children showed a DNAm pattern on the X chromosome that was intermediate between the normal male and normal female patterns (Fig. 1b). Together, this was suggestive of female blood being mixed with the cord blood of the newborn males, which could have occurred across the placenta during labor or after delivery.

Investigation of the cord blood collection procedure revealed that maternal contamination of the resulting cord blood after delivery was the most likely hypothesis to explain these unexpected DNAm patterns. With this insight, we then divided samples into three groups based on principal component 2 (PC2) of the full data and DNAm at cg05533223 on the X chromosome. As initially observed, PC2 clearly separated male from female samples, but was not associated with the major variables in the sub-study, ethnicity (ANOVA *p* > 0.8) or trauma exposure (*t* test *p* > 0.3). The CpG used, cg05533223, in the X-inactivation specific transcript (XIST) should be highly methylated in males and ~50% methylated in females [18]. Based on these two criteria, 17 males were contaminated (C), 64 were not contaminated (NC) and 5 were unclear (U) (Additional file 1: Figure S1 in Additional file 1). As we relied on X chromosome methylation levels, which would not differ between XX mothers and their XX daughters, this method was only applicable to XY male children. Since it called approximately 20% of male samples contaminated, we hypothesized that a similar proportion (approximately 13/64) of female children would also be contaminated. There was no reason to expect that the amount of maternal contamination due to sample collection would differ by sex, as all collection occurred in the same hospital using the same standard procedures.

### Using epigenetic age and genotyping no-calls to identify contaminated samples

We thus sought a way of discriminating contaminated females using other data. First, we tested epigenetic age by comparing the C and NC male samples using published methods [19]. As epigenetic age of cord blood samples has been demonstrated to be below 1 year, we hypothesized that mixing with maternal blood would result in an increase in epigenetic age of the whole sample. Though the DNAm age means were significantly different between C and NC, (two-sided Student’s *t* test *p* = 0.025), the large confidence intervals (−14.714880 to −1.077678) meant that this was not a sufficiently accurate test, despite the identification of at least 4 females who were likely contaminated (Additional file 1: Figure S2A). Using a similar method that estimates gestational age from DNAm data, we found similarly poor predictive value (Additional file 1: Figure S2B) [20].

Next, we used genotyping data to see whether a higher number of “no calls” from the Illumina PsychChip was associated with contamination. Our rationale was that mixing two blood samples together, even if genetically related, would result in a higher number of un-callable genotypes with signals falling between the three normal genotype groups. While performing better than epigenetic age, the extreme confidence intervals (34,281.73–10,811.97, *p* value <0.001), difference in basal number of no calls between males and females, and potential lack of genotyping data in other studies meant, in our opinion, this was not a suitable discriminatory screen either (Additional file 1: Figure S2C).

### Identification of CpGs indicative of contamination

We next reasoned that since DNAm has been shown to be highly different between neonates and adults, it might serve to discriminate contaminated samples. Using linear modeling followed by a random forests approach, we determined that 10 CpGs could discriminate between contaminated and non-contaminated male samples at 99% confidence (Additional file 1: Figure S2A, Additional file 1: Table S2). Importantly, the calculated thresholds for identifying contaminated samples were sensitive to normalization method, and so we present thresholds for two common normalization methods; SWAN and BMIQ [21, 22].

**Fig. 2:**
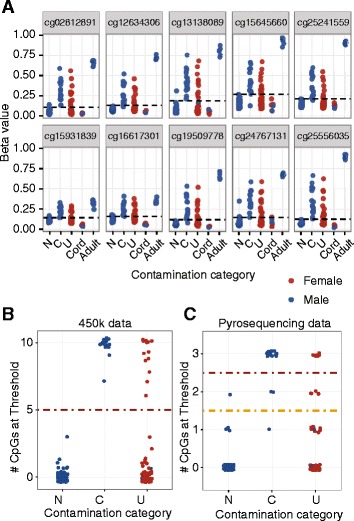
DNA methylation at 10 autosomal CpGs was sufficient to correctly identify all known contaminated male samples, and found 13 contaminated female samples. **a** The 10 CpGs selected by the random forest method clearly separate cord and adult samples, and also clearly discriminate non-contaminated (*N*) from contaminated (*C*) male samples, and divide unknown (*U*) samples into two groups. **b** Counting the number of sites over thresholds per sample (x axis), contamination was called if at least 5 of the 10 CpGs were above the threshold. Unclear males were all non-contaminated, and 13 females were identified as being contaminated. **c** A subset of 3 out of the 10 CpGs can be used for pyrosequencing screening. Two thresholds are shown—one requiring two of the three CpGs to be above the threshold to be called contaminated (*yellow*), and one requiring all three (*red*)

To identify the contaminated female samples, we applied the thresholds of these 10 CpGs to all of our samples (Fig. 2b). This method identified 13 females as contaminated, including the 4 previously identified by epigenetic age, in line with the approximately 20% expected based on proportion of contaminated males, and all 5 unclear males were categorized as non-contaminated (Fig. 2b). This showed that these 10 CpGs were sufficient for screening previously generated DNAm data to identify maternal blood contamination in male and female children. However, we wished to refine this panel so that samples could be screened prior to being run on an array in cases where contamination might be expected.

### Verification of screening CpGs using pyrosequencing

**Fig. 3:**
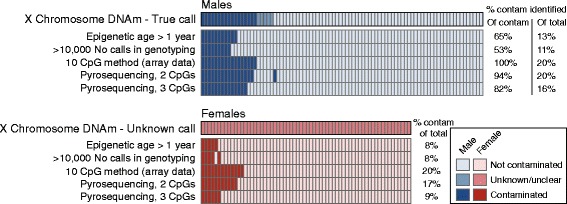
Summary of performance of all methods used to predict cord blood contamination. Each column represents the same participant across each method. The 10 CpG method using 450K array data was the most reliable, but using a subset of three CpGs was sufficient to identify at least 82% of contaminated samples

To ensure that this pre-screening method was quick and cost-effective, we focused on pyrosequencing and reduced the 10 identified CpGs to 3. These three CpGs had the best discrimination between contaminated and non-contaminated male samples and were sites for which a robust pyrosequencing assay could be designed (Table S2). After selecting cg25556035, cg15931839, and cg02812891, we performed pyrosequencing of these 3 sites on our original 150 samples (Fig. 2c). Interestingly, the assay that measured cg02812891 also measured cg13138089 as these CpGs are in close proximity. As these two CpGs were strongly correlated (*r* = 0.977) within the assay, we deemed cg13138089 to be redundant for the purpose of designing a minimal screen, though other groups may consider its inclusion in the screening process. A strict cut-off requiring all 3 CpGs to surpass the contamination threshold identified 14 male samples as contaminated, all consistent with the array and X chromosome data. A less stringent cut-off of 2 CpGs identified 17 male samples, with 1 false positive and 1 false negative. In females, the less stringent 2 CpG cut-off predicted 11 of the 13 samples called contaminated using the 450K array data, and the strict method predicted 6; neither had false positives. While this screen is not as accurate as the 10 CpG method from the 450K array data, it is sufficient to identify and eliminate the worst contaminated samples. All prediction methods and results are summarized in Fig. 3.

### Validation on second data set

**Fig. 4:**
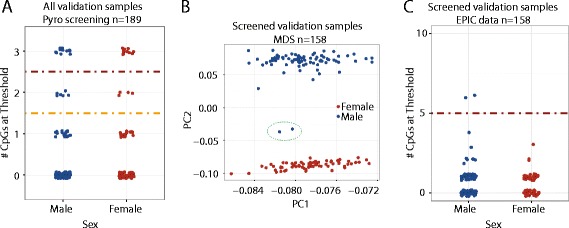
Pre-screening using the pyrosequencing method correctly identified contaminated male samples. **a** Applying a cut-off of 2 CpGs above the threshold (*yellow line*) to the 3 CpG pyrosequencing method on validation data, 18 males and 15 females were identified as contaminated. **b** Principal component plot of EPIC DNA methylation data on all non-contaminated samples with two male samples that had been called contaminated by pyrosequencing showed that contaminated male samples had been correctly identified. **c** Using the 10 CpG method from EPIC data, only the 2 male samples known to be contaminated had more than 5 CpGs above the threshold (*red line*)

To validate this screening method, 189 additional samples from the same cohort study were screened using the pyrosequencing assays. Eighteen males and 15 females were identified as contaminated using the 2 CpG cut-off, again approximating the 20% contamination rate we initially observed (Fig. 4a). We ran all 156 uncontaminated samples and 2 contaminated male samples on the EPIC array. We chose male samples as validation, as we could use sex-specific differences in DNA methylation at XIST on the X chromosome as independent confirmation of our screening method. Initial principal components plots showed that only the two known contaminated male samples demonstrated the intermediate DNAm pattern indicative of contamination (Fig. 4b). We then examined the 10 CpGs identified in our discovery data set and, as expected, only the 2 known male samples were identified as contaminated (Fig. 4c). This supports that 3 CpGs are sufficient to correctly eliminate contaminated samples prior to running on an array.

### Validation on publicly available data

**Fig. 5:**
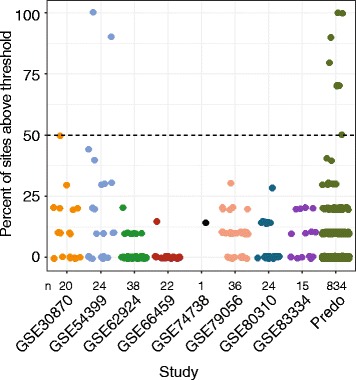
Identification of studies with significant contamination levels in public data. Using available data, we examined the 10 CpGs chosen to identify contamination, though some studies had previously filtered their data and some CpGs were not available. We called maternal contamination of samples if more than 50% of the available CpGs were above our contamination thresholds, and identified two studies (GSE54399 and PREDO) with contaminated samples

To address the frequency with which maternal blood contamination occurs in DNAm studies, we used nine published cord blood DNAm data sets (GSE30870, GSE54399, GSE62924, GSE66459, GSE74738, GSE79056, GSE80310, GSE83334, and PREDO). We applied our post hoc maternal contamination assay with 10 CpGs across these studies and identified 2 data sets with contaminated samples (Fig. 5). GSE54399 had 2/24 (~10%, 1 male and 1 female) samples indicating contamination, and PREDO 8/834 (~1%, 4 males and 4 females). Across all studies, maternal blood contamination was present at a frequency of approximately 1% (10/1014), but the study-specific pattern suggests that contamination may be related to specific collection methods.

Finally, we examined our discovery samples, validation samples, and the publicly available data together to determine whether our 10 CpG method was affected by batch or technology. We compared the residuals of each sample’s methylation to thresholds of each of our 10 CpGs (Additional file 1: Figure S3). We observed similar distributions for each CpG in all studies except for the validation cohort, the only one to use the EPIC array. These data were normalized with methods consistent with the GEO data, so the effect is due to technology and not normalization method. This suggests that, despite successfully identifying the known contaminated samples in our EPIC cohort, the 10 CpG method is influenced by array technology and thus using all 10 CpGs is highly recommended when working with EPIC data.

## Discussion

The popularity of cord blood collection for both research and medical purposes means that it is more important than ever to ensure that the collected blood is free of contaminating maternal white blood cells. In this study, we initially observed unusual patterns in a pre-normalization MDS plot driven by X chromosome DNAm in male cord blood samples. After consulting the collection procedure, we strongly suspected that maternal blood contamination was present in a subset of the cohort. We developed a universal screen for identifying maternal contamination of cord blood using DNAm at a subset of CpGs in the genome. This screen can be applied to already-generated DNAm data from the 450K or EPIC microarray platforms, but perhaps more interestingly, simple pyrosequencing at a subset of CpGs was highly efficient at identifying contaminated samples. This approach could then be used to screen DNA from samples destined for many purposes, including genotyping or gene expression methods or even cord blood banking.

The described methods can reliably detect maternal blood contamination at levels that would confound genetic or epigenetic analyses. The amount of contamination observed in all three studies could interfere with DNAm data analysis, but our proposed 10 CpG post hoc screen accurately identified and removed contaminated male and female samples. The three CpG pyrosequencing screen will be useful primarily for: (a) cord blood that is not destined for DNAm assessment, such as genotyping or gene expression studies, (b) when the expected rate of contamination is high, or (c) if it is particularly disadvantageous to run a possibly contaminated sample. Our method has significant advantages compared to other methods of detection of maternal contamination. For example, FISH requires whole cells, and most TaqMan assays require DNA samples from both mother and child [5–8, 11, 23]. For our DNAm-based detection of contamination, neither is required, however, this does mean that we were not able to benchmark our method against these others, as we did not have the required sample types.

While standard procedures exist for the collection of cord blood, our results suggest that maternal contamination is still observed. In our cohort study, the rate of contamination was 20%, and we observed two other studies with appreciable levels of contamination, at 10% and 1% of samples. This suggests that maternal contamination is considerable overall, but importantly might occur more frequently in some studies. Our samples were collected from rural communities in a region near Cape Town, South Africa, and the publically available study with the highest ratio of contaminated samples (GSE54399) was collected in the Congo [24]. Collection procedures used in studies with less experience, many collections per day, or with fewer resources may be more prone to introducing maternal contamination in cord blood.

As our study used real collected cord blood samples, it is difficult to estimate the specific detection limit of our screening method. Since the differences in DNAm are proportional to the amount of contamination, any samples that fail to meet the recommended cut-offs must contain at most a small contribution of maternal blood. This uncertainty is reflected in our attempt to use either epigenetic age or number of no calls in genotyping data to screen for maternal contamination. Both methods identified some but not all contaminated samples, and had very high variability. It is thus unclear whether these methods are inherently less predictive than the 10 CpGs we identified, or if the amount of contamination in our samples was too small to detect by these methods. To determine exact proportions of contamination detectable by these methods, a follow-up study may consider creating known dilutions of cord blood spiked with maternal blood, and assessing epigenetic age, genotyping no calls, as well as our 10 and 3 CpG methods. Thus while our proposed method cannot guarantee that all maternal contamination is eliminated, it should assure that the most contaminated samples are identified and that any remaining contamination has a minimal impact on downstream applications.

Finally, given that we recognized the contamination issue during routine quality control, it is possible that many researchers already find and remove some contaminated samples from their cord blood DNAm studies. However, our inability to identify contaminated female samples during QC and the fact that we detected contaminated samples in published data demonstrate that normal QC is not sufficient to completely eliminate contamination, particularly of female samples. The 10 CpG panel is then useful to ensure the removal of any contaminated samples once DNAm data has been generated.

## Conclusions

In conclusion, we have created a screen to test for maternal contamination in cord blood that has two independent applications: first, a simple and cost-effective method to screen DNA from cord blood using pyrosequencing, and second, a way to identify contaminated samples post hoc from DNAm arrays. Both clinicians and researchers should be aware of the possibilities of cross-contamination of maternal and cord blood, and the CpGs we have identified will allow for easy identification and removal of contaminated samples.

## Methods

### Cord blood collection

In the Drakenstein study, cord blood was collected by trained staff after delivery of the baby but before delivery of the placenta. The cord was clamped and cut, then the clamp was released and cord blood drained by gravity into a kidney dish, then collected using a syringe for processing and storage.

Samples used in this analysis were selected from the full Drakenstein cohort for a sub-study on exposure to maternal traumatic stress, and approximately 30% of children had been exposed to maternal trauma. The Drakenstein cohort general inclusion criteria are described elsewhere [25]. Study participants with available neuroimaging data were preferentially selected where feasible. Only samples of offspring whose mothers had provided informed consent for the collection, storage, and future analyses of DNA were eligible for inclusion.

### DNA methylation data

In the discovery data set, DNAm was measured on 150 samples (86 males, 64 females) using the Illumina Infinium HumanMethylation450 bead array (Illumina, San Diego, USA), per manufacturer’s instructions and previous work [26]. Next, we imported the raw data into Illumina GenomeStudio Software for background subtraction and color correction, then exported it for processing using the lumi package in R (version 3.2.3) [27]. Initial quality control and identification of maternal contamination in male samples by multi-dimensional scaling (MDS) plotting and X chromosome DNAm occurred prior to removal of any probes. We then removed rs probes, X and Y chromosome probes, probes with detection *p* values above 0.05, probes with less than three beads contributing to signal, and previously identified cross-reacting probes, for a total of 421,993 probes remaining [28]. Quantro analysis indicated that quantile normalization was allowable, so we first normalized with the lumi quantile method, then with SWAN for probe type correction [21]. Finally, we used ComBat to remove chip and row effects [29].

For validation data, analysis was identical with three exceptions: first, data were generated using the Infinium HumanMethylationEPIC (Illumina, San Diego, USA) on 158 samples (89 males, 69 females). Second, we used BMIQ normalization, and only performed ComBat on the chip effects [22]. Third, we only retained the 10 probes identified as indicators of contamination.

Publicly available data were downloaded from GEO (GSE30870, GSE54399, GSE62924, GSE66459, GSE74738, GSE79056, GSE80310, and GSE83334), pre-processed as above, and data from the PREDO study were provided by coauthors [30].

### Genotyping data and no calls analysis

Genotyping data were generated using the Illumina PsychChip (Illumina, San Diego, USA) per manufacturer’s instructions then raw data were imported into GenomeStudio using the PsychChip cluster file. Genotypes were called by default methods in the GenomeStudio software by comparing the sample intensities at each locus to expected genetic clusters, and a default quality metric represented a sample’s distance from the expected cluster. The standard cut-off of 0.15 was used to establish a threshold, outside of which samples were too far from the cluster and the GenomeStudio software did not call a genotype at that locus. *p* values and 90% confidence intervals for differences between contaminated and non-contaminated samples were assessed using two sided Student’s *t* test with the t.test function in R statistical software [27].

### Epigenetic and gestational age analysis

Epigenetic age was determined using two epigenetic clocks, one which outputs chronological age and is designed for adults, and the other which outputs gestational age and is designed for newborns [19, 20]. Both methods use a panel of CpGs whose collective DNA methylation status is strongly predictive of chronological age. As above, *p* values and confidence intervals for the difference between contaminated and non-contaminated samples was calculated using two sided Student’s *t* test with the t.test R package [27].

### Identification of sites used to detect contamination

To discover CpGs capable of identifying maternal contamination, we first performed linear modeling on whole cord (GSE## to be determined) and adult (Flow.sorted.blood.450K R package) blood DNAm data to identify sites that were most different between cord and adult blood [31, 32]. With thresholds of adjusted *p* value <1 × 10^−20^ and mean beta value difference greater than 0.2, we identified 2250 DNAm sites that were differentially methylated between cord and adult. Though these sites were all statistically significant, they were redundant in their multiplicity, and we wished to reduce the number of sites to make assessment more feasible. Thus, we analyzed this large set of 2250 CpGs with a random forest approach from machine learning [33]. This ensemble learning method is designed to take advantage of multiple predictors, while also addressing small-sample over-fitting. The random forest method ranked the DNAm sites by mean decrease in accuracy, a measure of their importance. We then applied binary recursive partitioning to choose the threshold values separating contaminated from non-contaminated samples [34].

### Pyrosequencing verification

We used PyroMark Assay Design 2.0 (Qiagen, Inc.) software to design bisulfite pyrosequencing assays covering three identified CpGs (sequences in Additional file 1: Table S1). DNA was bisulphite converted using the EZ DNA Methylation Kit (Zymo Research), and PCR and pyrosequencing performed as previously described [35]. Streptavidin-coated sepharose beads were bound to the biotinylated strand of the PCR product and were then washed and denatured to yield single-stranded DNA. Sequencing primers were then added for pyrosequencing per manufacturer’s instructions (Pyromark™ Q96 MD Pyrosequencer, Qiagen, Inc.).

## Additional file

Additional file 1:
**Table 1.** Primer sequences used for pyrosequencing. **Table 2.** Beta value thresholds used for DNA methylation arrays and pyrosequencing. **Figure S1.** Strategy used to identify contamination in male samples. Plotting PC2, which separated male from female samples in our data, against DNA methylation at a CpG in XIST on the X chromosome, revealed three populations of male samples: contaminated, non-contaminated, and a group of five samples which were unclear. **Figure S2.** Neither epigenetic age (A), gestational epigenetic age (B) nor number of genotyping “no calls” (C) were sufficient to identify maternal blood contamination of cord blood. In all cases, contaminated and non-contaminated males showed high overlap, indicating insufficient discrimination. **Figure S3.** Across-batch differences in DNA methylation level support the use of multiple predictive CpGs for identification of contamination. Residual plot of discovery data (A), validation data (B), publically-available data (C), and PREDO (D) indicate technical spread across samples and studies. In particular, EPIC data (second cohort, top right) shows greater variability and higher baseline levels than the 450K data sets. (PDF 759 kb)

## Abbreviations

DNAm: DNA methylation
FISH: Fluorescent in-situ hybridization
GEO: Gene expression omnibus
MDS: Multi-dimensional scaling
PCR: Polymerase chain reaction
