# Supplementary material for: Maternal blood contamination of collected cord blood can be identified using DNA methylation at three CpGs
Source: Clin Epigenetics. 2017 Jul 25;9:75. doi: 10.1186/s13148-017-0370-2 (PMC5526324; doi:10.1186/s13148-017-0370-2)
Supplement: Additional file 1: — Table 1. Primer sequences used for pyrosequencing. Table 2. Beta value thresholds used for DNA methylation arrays and pyrosequencing. Figure S1. Strategy used to identify contamination in male samples. Plotting PC2, which separated male from female samples in our data, against DNA methylation at a CpG in XIST on the X chromosome, revealed three populations of male samples: contaminated, non-contaminated, and a group of five samples which were unclear. Figure S2. Neither epigenetic age (A), gestational epigenetic age (B) nor number of genotyping “no calls” (C) were sufficient to identify maternal blood contamination of cord blood. In all cases, contaminated and non-contaminated males showed high overlap, indicating insufficient discrimination. Figure S3. Across-batch differences in DNA methylation level support the use of multiple predictive CpGs for identification of contamination. Residual plot of discovery data (A), validation data (B), publically-available data (C), and PREDO (D) indicate technical spread across samples and studies. In particular, EPIC data (second cohort, top right) shows greater variability and higher baseline levels than the 450K data sets. (PDF 759 kb) [file 13148_2017_370_MOESM1_ESM.pdf]

**Tables**

Table 1: Primer sequences used for pyrosequencing

| Primer name | Sequence |
|---|---|
| **cg25556035 F** | AGTTTTTGTTTTTTAGGGTAGGTATTGATA |
| **cg25556035 R-biot** | AACTCCAACACAAACTATTTACAC |
| **cg25556035 Seq** | GTTTTTTTTTTTTTTTAGTATTAATTAATTTAAG |
| **cg15931839 F** | TAGATTTTATTTTAAAGTTAGGGTGTGTGG |
| **cg15931839 R-biot** | AACCAAACCATATTTAAAACCTTATTC |
| **cg15931839 Seq** | GAATAGAAAAAAATTAGTTAGGAG |
| **cg002812891 F-biot** | ATGGTGTAGTAGGTGAGTTTGT |
| **cg002812891 R** | TACAACCCACCTCTACTCCATCCTAAC |
| **cg002812891 Seq** | AACTACCACCCCATACCATCCC |

Table 2: Beta value thresholds used for DNA methylation arrays and pyrosequencing

| | Array-SWAN | Array-BMIQ | Pyrosequencing |
|---|---|---|---|
| **cg25556035** | 0.123726 | 0.085192 | 0.057375 |
| **cg13138089** | 0.18849 | 0.214598 | |
| **cg12634306** | 0.128539 | 0.140893 | |
| **cg25241559** | 0.209688 | 0.230536 | |
| **cg02812891** | 0.107235 | 0.102181 | 0.029425 |
| **cg15645660** | 0.267049 | 0.279702 | |
| **cg19509778** | 0.117568 | 0.062266 | |
| **cg15931839** | 0.14165 | 0.084144 | 0.081525 |
| **cg16617301** | 0.159146 | 0.106105 | |
| **cg24767131** | 0.145945 | 0.185700 | |

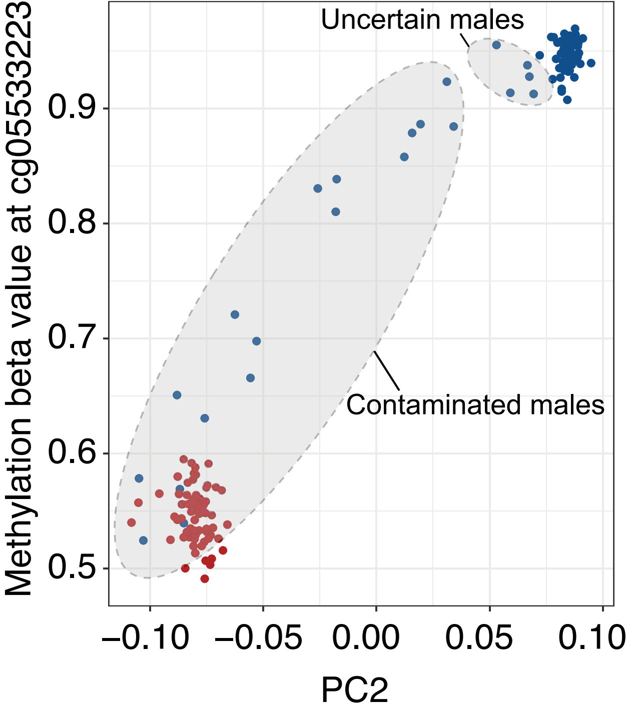

Uncertain males
Contaminated males
Methylation beta value at cg05533223
0.9
0.8
0.7
0.6
0.5
−0.10
−0.05
0.00
0.05
0.10
PC2

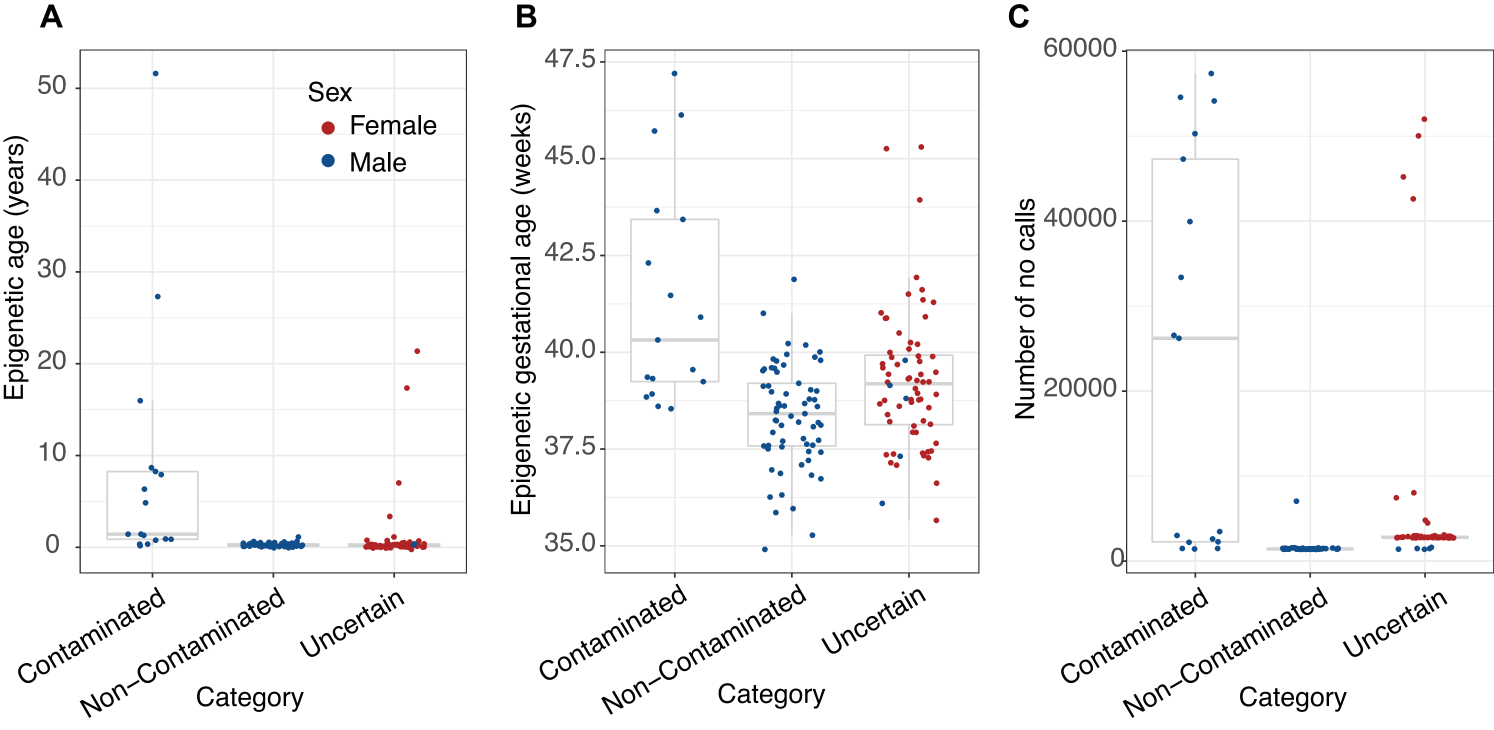
A
B
C
Sex
Female
Male
Epigenetic age (years)
50
40
30
20
10
0
Epigenetic gestational age (weeks)
47.5
45.0
42.5
40.0
37.5
35.0
Number of no calls
60000
40000
20000
0
Contaminated
Non–Contaminated
Uncertain
Category

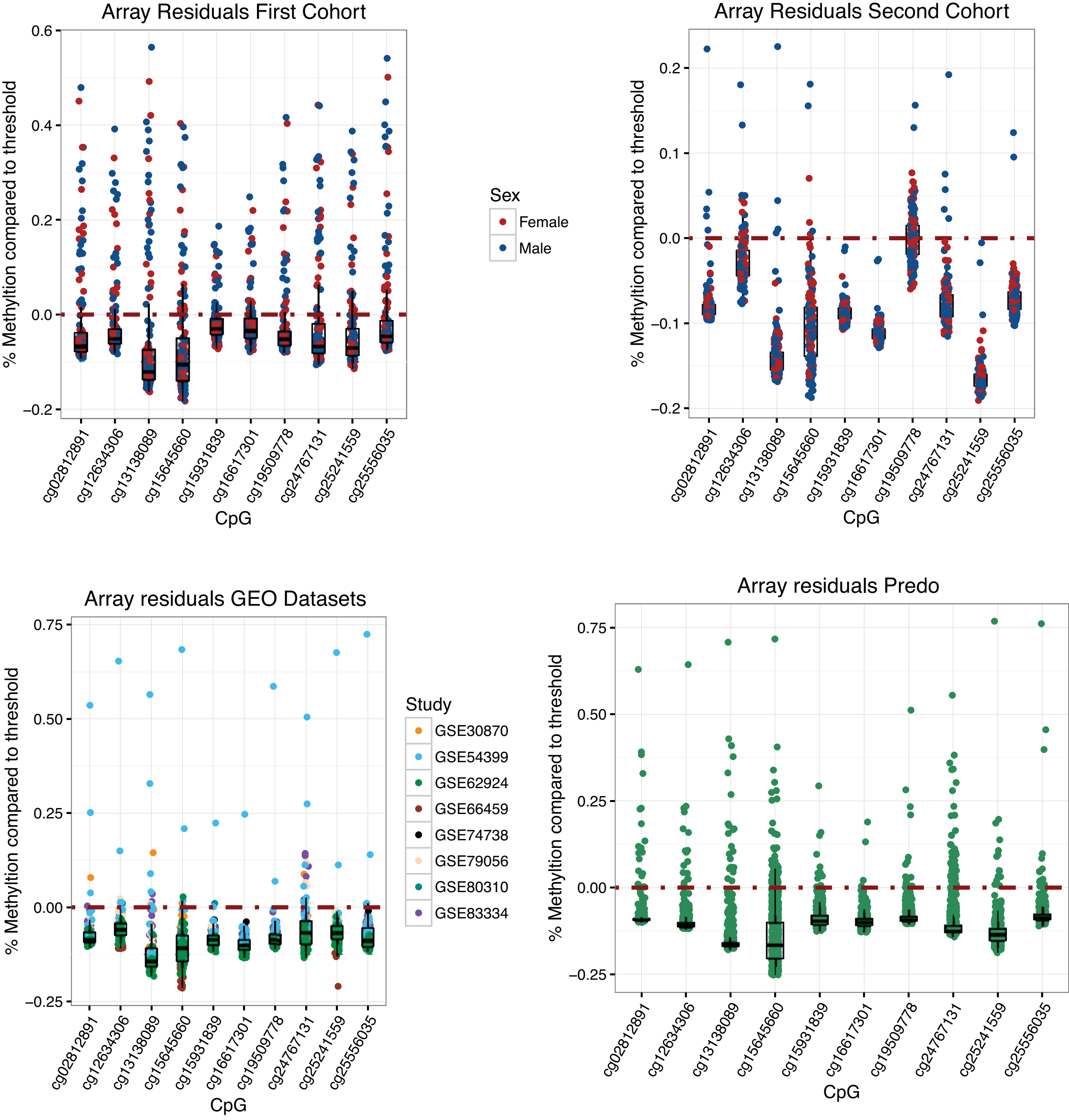

Array Residuals First Cohort
% Methyltion compared to threshold
CpG
Sex
Female
Male
Array Residuals Second Cohort
Array residuals GEO Datasets
Study
GSE30870
GSE54399
GSE62924
GSE66459
GSE74738
GSE79056
GSE80310
GSE83334
Array residuals Predo
cg02812891
cg12634306
cg13138089
cg15645660
cg15931839
cg16617301
cg19509778
cg24767131
cg25241559
cg25556035
